# Effect of Preheating on the Residual Stress and Material Properties of Inconel 939 Processed by Laser Powder Bed Fusion

**DOI:** 10.3390/ma15186360

**Published:** 2022-09-13

**Authors:** Martin Malý, Klára Nopová, Lenka Klakurková, Ondřej Adam, Libor Pantělejev, Daniel Koutný

**Affiliations:** 1 Institute of Machine and Industrial Design, Faculty of Mechanical Engineering, Brno University of Technology, Technická 2896/2, 616 69 Brno, Czech Republic; 2 Institute of Materials Science and Engineering, Faculty of Mechanical Engineering, Brno University of Technology, Technická 2896/2, 616 69 Brno, Czech Republic; 3 Central European Institute of Technology (CEITEC), Brno University of Technology, Purkyňova 656/123, 612 00 Brno, Czech Republic

**Keywords:** laser powder bed fusion, selective laser melting, Inconel 939, preheating, residual stress

## Abstract

One of the main limitations of laser powder bed fusion technology is the residual stress (RS) introduced into the material by the local heating of the laser beam. RS restricts the processability of some materials and causes shape distortions in the process. Powder bed preheating is a commonly used technique for RS mitigation. Therefore, the objective of this study was to investigate the effect of powder bed preheating in the range of room temperature to 400 °C on RS, macrostructure, microstructure, mechanical properties, and properties of the unfused powder of the nickel-based superalloy Inconel 939. The effect of base plate preheating on RS was determined by an indirect method using deformation of the bridge-shaped specimens. Inconel 939 behaved differently than titanium and aluminum alloys when preheated at high temperatures. Preheating at high temperatures resulted in higher RS, higher 0.2% proof stress and ultimate strength, lower elongation at brake, and higher material hardness. The increased RSs and the change in mechanical properties are attributed to changes in the microstructure. Preheating resulted in a larger melt pool, increased the width of columnar grains, and led to evolution of the carbide phase. The most significant microstructure change was in the increase of the size and occurrence of the carbide phase when higher preheating was applied. Furthermore, it was detected that the evolution of the carbide phase strongly corresponds to the build time when high-temperature preheating is applied. Rapid oxidation of the unfused powder was not detected by EDX or XRD analyses.

## 1. Introduction

Additive manufacturing (AM) technologies have been known since the 1980s and since then have made a great leap toward the reliable production of near net shape components made from a wide range of materials [1,2]. The ability to produce complex geometries even from materials that are to be processed opens new possibilities for increasing machine efficiency and producing organically shaped components [3]. The last 20 years have led to rapid progress in metal AM technologies, where the most studied and used are powder bed fusion (PBF) technologies [4,5,6]. PBF uses for component production an energy source which selectively fuses layers of fine metal powder according to part geometry until the components are completely finished. A laser beam is more commonly used as an energy source than an electron beam. Laser powder bed fusion (LPBF) takes the largest share among research and industrial applications, with more than 80% compared to electron beam melting (EBM) due to lower machine and operating costs and better beam resolution [7]. Research initiatives have focused on the introduction of new materials, repeatability of the process, qualification standards, and the reduction of production costs, which hinder the acceptance of the technology in many industries [8]. Currently, a wide range of materials can be processed, including aluminium alloys, stainless steel, titanium alloys, and nickel-based superalloys [4].

Nickel-based superalloys are used in the energy and aerospace sectors because of their excellent oxidation resistance and creep properties at high temperatures. Traditionally, a casting process has been used to produce near net shape components. However, processing by PBF offers greater complexity, making it a tempting manufacturing process for nickel-based superalloys [9]. Inconel 718 (IN718) is the most widely used and studied nickel-based superalloy in PBF and is suitable for applications in the temperature range up to 650 °C [10]. For applications at temperatures up to 850 °C, another alloy must be used, for example, Inconel 939 (IN939) [11]. IN939 is a high-chromium alloy that can be used at elevated temperatures for a long period of time due to the strengthening of gamma prime (γ’-Ni_3_(Al, Ti, Nb)) precipitates and carbides [12]. 

Components produced by PBF have a fine microstructure and exhibit static mechanical properties comparable to or even higher than their conventionally produced counterparts [4]. However, to achieve high mechanical properties, the correct process parameters for fabrication must be set. A non-optimal process setting can lead to gas porosity, key hole porosity, or lack of fusion defects [13]. In addition, defects such as cracks and part deformations have also been found in LPBF as a result of inhomogeneous heating and large thermal gradients, which lead to the evolution of anisotropic residual stress (RS). RSs are stresses that remain in material when equilibrium with the surrounding environment is reached [14]. The three types of RSs can be distinguished according to the scale on which they act [14]. Type I of RSs operate on macroscopic scales, and when their magnitude exceeds the yield strength of the material, part deformations may occur. Type I RSs are the most studied and described in the literature because they directly affect the manufacturability of the geometry and can be influenced by process parameters [8,15]. Although heat treatment for stress relieve can be performed after the production run, RS evolution occurs even during production and causes failures such as warping. Therefore, parts must be attached to the build platform directly or via support structures that must be fabricated along with the parts. Support structures increase manufacturing time, material consumption, and post-processing costs. However, RSs of the type I can be beneficial when they are applied on the finished component surface; for example, by shoot peening, they can increase fatigue resistance [16]. Type II RSs operate on the individual grain scale as a result of the different thermal and elastic properties of the differently oriented grains [14]. Type III RSs are on the atomic scale as a result of crystallographic lattice disorders. Types II and III are difficult to measure and are less important in terms of their mechanical properties [17]. Therefore, only type I of RSs are discussed in the following text. The problem with RS is more common in LPBF than in EBM, which is due to the higher preheating temperatures of the build chamber (500–800) °C in EBM. Higher preheating temperature reduces the temperature gradients and cooling rate [18].

Many researchers have investigated the proper settings of process parameters in LPBF for RS mitigation and have attempted to find universal laws for RS reduction [15,19]. The formation of RS has also been studied as a function of material properties. Many studies have shown that materials with higher thermal diffusion and thermal conductivity have a higher RS [8]. The RSs were also higher in materials with higher ultimate strength, yield strength, and coefficient of thermal expansion. However, the magnitude of RS also depends on the used process parameters and each material requires an optimum setting for the production of homogeneous parts [20]. Therefore, such universal laws are not always valid and inconsistencies can be found in the literature [3,21]. Such inconsistencies are also observed in the preheating of the base plate, where a higher preheating temperature generally reduces the RS [22], but excessive heating can increase RS [23]. Therefore, the optimal setting must be determined for each material. Excessive preheating of the powder bed can also have a negative effect on the properties of unfused powder and limit its recycling [15,24]; thus, it needs to be checked.

As outlined here, the evolution of RS in LPBF limits the manufacturing freedom, dimensional accuracy, and increases post-processing costs. Therefore, it is important to investigate the possibilities of mitigating RS through in-process process parameters and to develop optimal conditions that maintain it at a minimum level. This study investigates the effect of high-temperature preheating of the base plate up to 400 °C on the evolution of RS in IN939 produced by the LPBF. Moreover, it contains complex analyses describing the preheating effect on the microstructure, tensile properties, hardness, and properties of the unused powder, thus providing comprehensive information about material behavior under high-temperature preheating conditions.

## 2. Materials and Methods

### 2.1. Powder Characterization

**Figure 1:**
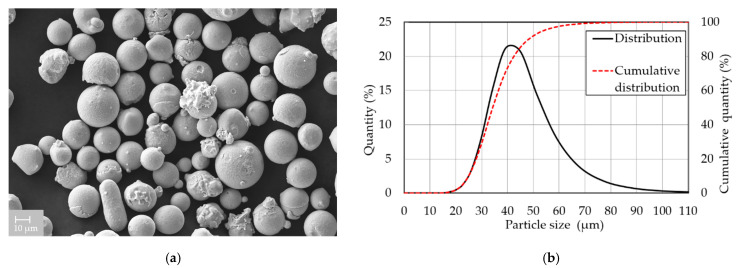
IN939 powder characterization: (**a**) SEM image showing mostly spherical particles; (**b**) particle size distribution.

Gas atomized IN939 powder (SLM Solutions Group AG, Lübeck, Germany) was used in this study. The chemical composition delivered by the material supplier was verified by energy dispersive X-ray spectroscopy (EDX) performed on AZtec SW (Oxford Instruments plc. UK, Abingdon, UK) and LYRA3 XMH (TESCAN, Brno, Czech Republic) scanning electron microscope (SEM) accessories. EDX analysis performed on three specimens showed that the mean values of the chemical composition met the alloy composition requirements, except for Cr, whose composition was 2 wt. % below the standard (Table 1). SEM analysis detected mainly spherical powder particles (Figure 1a). The particle size distribution (Figure 1b) was checked using laser diffraction analyzer LA-960 (Horiba, Kyoto, Japan). The median size was 34.3 μm and the mean particle size was 35.8 μm. The diameter of 10% of the particles was smaller than 24.7 μm, while the diameter of 90% did not exceed 48.7 μm.

X-ray diffraction (XRD) was performed on a SmartLab diffractometer (Rigaku, Austin, TX, USA) using CuKα radiation. The device operated with voltage V = 40 kV and current A = 30 mA. Continuous scanning was performed for 2θ from 35° to 120° with a step size of 0.02°. D-Tex with a β-filter in the secondary beam was chosen as the detector. PANAnalytical High Score Plus was used for qualitative and quantitative analyses using the pdf2 and ICSD databases. Quantitative analysis was performed using the Rietveld method with the external standard SI. Method error ±1 wt. %. The limit of detection was 2 wt. %.

### 2.2. Specimen Fabrication and Characterization

An SLM 280HL 3D printer (SLM Solutions Group AG, Lübeck, Germany), equipped with a 400 W ytterbium fiber laser YLR-400-WC-Y11 (IPG Photonics, Oxford, MS, USA) with a focus diameter of 82 µm and a Gaussian shape power distribution, was used to fabricate the specimens. An in-house manufactured heating device was used to preheat the stainless-steel base plate. The heating device uses resistive heating elements and the temperature is controlled by a PID regulator and thermocouples. The accuracy of the temperature on the base plate is ±20 °C. The base plate preheating cannot ensure homogeneous preheating for high specimens, and the surface temperature gradually decreases within the specimen height. However, for specimens up to 10 mm, the preheating temperature should not decrease more than 15 °C [25]. Argon was used as the protective gas. The O_2_ content was kept below 1000 ppm during the production run. The powder moisture content, which was checked using Hytelog (B + B Thermo-Technik GmbH, Donaueschingen, Germany) before each production run, was 3 ± 2%. The fabricated specimens were analysed in the as built state. 

**Figure 2:**
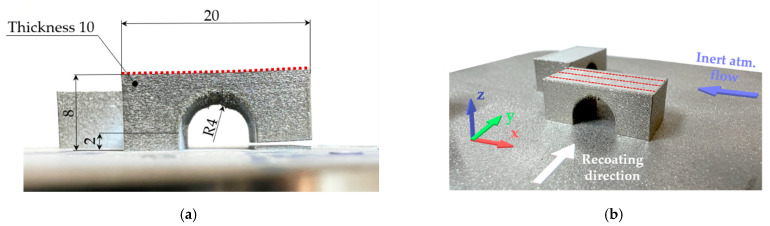
Bridge-shaped specimens: (**a**) specimen in the cut state with dimensions and marked top surface (red dotted line); (**b**) layout on the platform in the as built state with marked virtual section for measuring top surface distortion.

The amount of RS was estimated using the bridge curvature method (BCM), in which one of the two pillars of the bridge-shaped specimens was cut out by electro-discharge machining, and the deflection of the top surface was measured (Figure 2a). The BCM is widely used in PBF and allows a qualitatively asses process parameters and their direct effect on RS through deformation of the part [26,27]. The BCM specimens were produced directly on the build platform in the transverse (90°) and longitudinal (0°) directions corresponding to the direction of the powder recoating (Figure 2b). The geometry of the top surface was measured in each state using an optical 3D scanner Atos TripleScan 8M (GOM GmbH, Braunschweig, Germany). Data of the top surface deformation were determined as a mean value of the three virtual sections at a distance of 2.5 mm from each other. The virtual sections are shown in Figure 2b as red dotted lines on the top surface of one specimen.

The distortion of the top surface, relative density, macrostructure, microstructure, and hardness were determined on the BCM specimens. Two horizontal tensile test specimens were produced for each preheating temperature according to the DIN 50125 B 30 × 6 mm standard. Cylindrical shape specimens with a gauge length of 30 mm and diameter of 7.2 mm were fabricated using LPBF and their surfaces were turned to a standard diameter of 6 mm before tensile testing. The tensile test at room temperature was carried out on Zwick/Roell Z250 equipped with extensometer MultiXtens (Zwick Roell Group, Ulm, Germany). The strain rate was set to 0.00007 s^−1^ until the 0.2% proof stress was reached, then the strain rate was 0.001 s^−1^.

The BCM and tensile specimens were fabricated at room temperature (RT) and base plate preheating temperatures of 200 °C and 400 °C. The preheating temperature of 400 °C is the maximum reachable temperature of the heating device. The meander scanning strategy was used and other process parameters were set for 0.05 mm layer thickness according to the powder supplier’s recommendation. 

For material analyses, the BCM specimens that were aligned with gas flow were cut in the middle along the *Z*-axis, and the XZ and XY planes were analysed. The orientation of the specimens on the platform and the XYZ axis are shown in Figure 2b. The cut specimens were hot-pressed into a polymer resin, ground, with SiC abrasive papers up to 4000 grit size, and subsequently polished by 3 μm and 1 μm diamond paste. For SEM analysis, the specimens were additionally mechanochemically polished using Struers OP-S suspension. To evaluate relative density, overview images of the XZ planes were taken in two different specimen planes at least 1 mm apart using the 3D digital microscope Keyence VHX-6000 (Keyence, Mechelen, Belgium). ImageJ software was used to analyze the relative density using an automatic threshold. The resulting value of the relative density is the average of the two different XZ planes of the specimen.

The macrostructure and microstructure were examined in an etched state (Kalling’s: 2 g—CuCl_2_, 40 mL—HCl, and 60 mL—methanol) using an Olympus GX51 light microscope (Olympus, Tokyo, Japan) for macrostructure evaluation and SEM ZEISS Ultra Plus (Carl Zeiss AG, Oberkochen, Germany) and Tescan LYRA3 XMH (TESCAN ORSAY HOLDING, Brno, Czech Republic) for microstructure observation using backscattered electrons.

The width of the melt pool was determined in the XZ planes using a linear method in which the length of the lines used was divided by the number of intersecting melt pools. Furthermore, the mean columnar grain width was measured in the SEM images using linear method and ImageJ software. The mean values of the width of the melt pool and the columnar grain width are composed of at least 10 linear measurements. Moreover, the mean carbide size and occurrence per area were measured on SEM images using ImageJ software. The mean value of carbide size is made of a minimally 50 measurements. The value of occurrence per area was measured as the number of carbides in at least six areas with a size of 20 μm^2^.

A Vickers hardness test was performed to observe potential changes in the BCM specimen. The Vickers hardness test was performed in the XZ plane of the polished BCM specimens using a Qness testing machine Q10A (ATM Qness GmbH, Golling an der Salzach, Austria) with a test load of 9.807 N (HV 1) and holding time of 10 s, according to the ASTM E92-17 standard. The hardness on the XZ plane was measured in three lines along the Z-axis. On each line was measured 20 indentions homogeneously spread over the entire height of the sample. 

## 3. Results

The results presented in this chapter show the effect of base plate preheating on the BCM specimens fabricated using LPBF. First, the effect on the deformation of the BCM specimens in the cut state is shown, followed by the macrostructure and microstructure evaluation. Changes in the macrostructure and microstructure of the material led to changes in the tensile properties and hardness properties, which are confirmed by the measured data presented in this chapter. The IN939 powder was exposed to elevated preheating temperatures. Therefore, the chemical and phase composition were verified by EDX and XRD analysis to evaluate its reusability.

### 3.1. Effect of Powder Bed Preheating on Residual Stress

**Figure 3:**
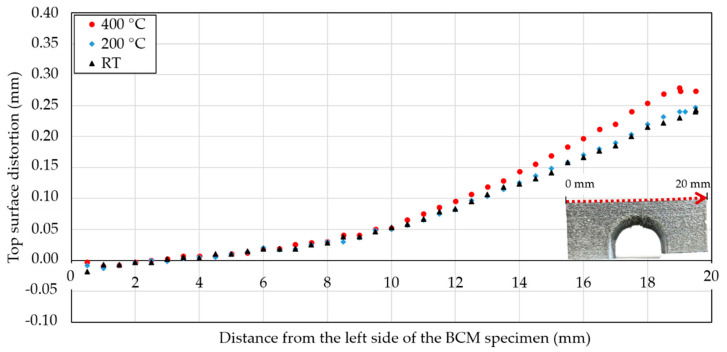
The mean values of 0° and 90° top surface deflection of BCM specimens according to base plate preheating temperature.

Powder bed preheating usually leads to a lower RS and therefore less part distortion [15,22,28]. In the case of the IN939 BCM specimens, higher preheating temperatures caused a greater deflection of the top surface (Figure 3). The top surface distortion did not change significantly when RT and preheating at 200 °C were applied. The maximum distortion of 0.24 mm was measured at RT, while at 200 °C, the maximum top surface distortion was 0.25 mm, which is an increase of 4.2%. However, preheating to 400 °C resulted in an increase in distortion of 16.2% compared to RT and the maximum measured distortion was 0.28 mm. The deflection data in Figure 3 are the mean values of two specimens built at 0° and 90° according to the recoating direction.

The orientation of the specimen on the build platform significantly affected the distortion of the top surface. Maximum distortion values on the top surface were always measured in the longitudinal (0°) direction according to the recoating of the powder (Figure 2a). The maximum deformation of the top surface of the longitudinal specimens was 0.1 mm greater (Table 2).

### 3.2. Effect of Powder Bed Preheating on Macrostructure and Microstructure

The influence of the base plate preheating temperature on the relative density was not significant. In all cases, the relative density was close to 100%. The relative density of the RT specimen was 99.9%, for specimens with a preheating at 200 °C (or 400 °C) the relative density was 99.9% (or 99.8%). The character and distribution of the pores were similar for all levels of the specimens and the studied planes. The observed pores had a spherical shape with diameters between 15 and 65 μm and were predominantly located near the surface of the specimens. No macrocracks and microcracks were observed in either case.

**Figure 4:**
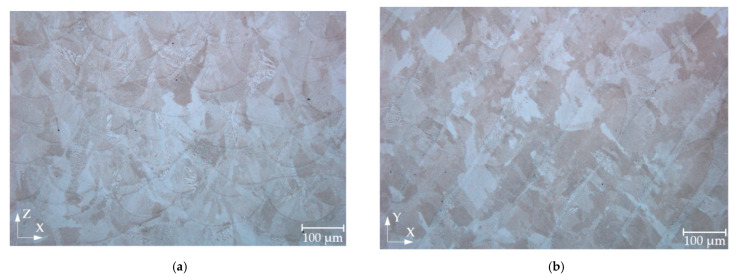
Light microscopy images of LPBF processed specimens fabricated using base plate preheating at 400 °C. (**a**) XZ plane. (**b**) XY plane.

**Figure 5:**
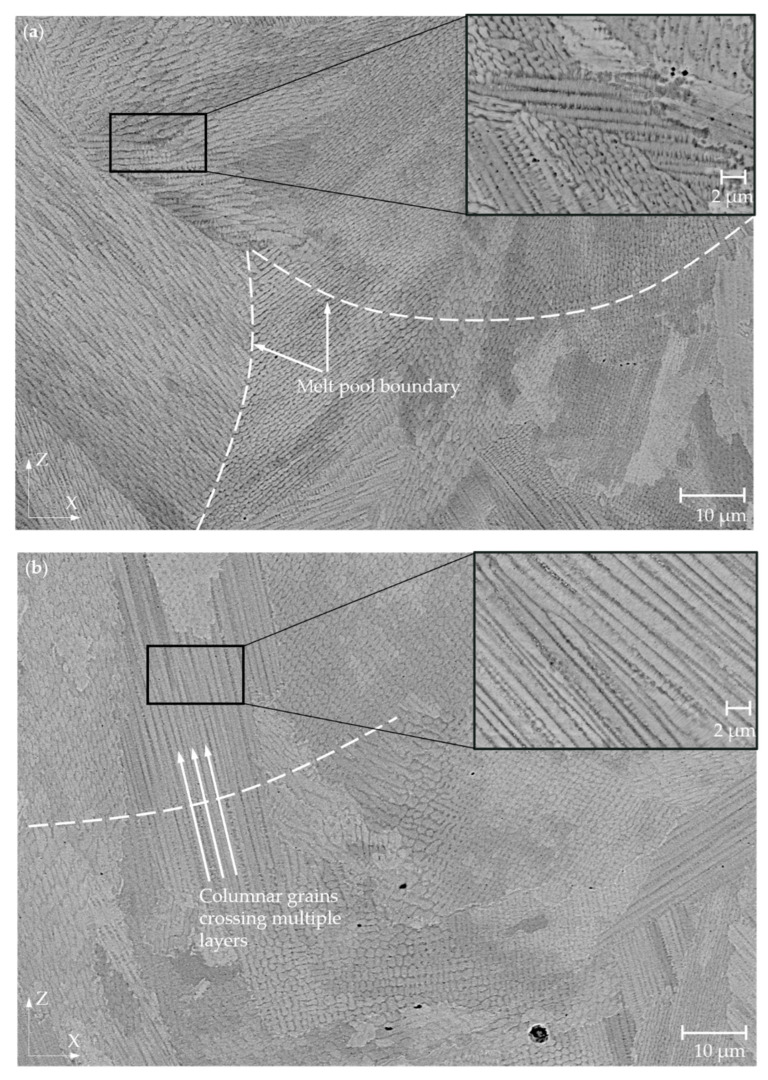
SEM-BSE microstructure of IN939 specimens fabricated at different preheating temperatures. (**a**) XZ plane of the RT specimen and (**b**) XZ plane of the specimen with a base plate preheated at 400 °C.

Light microscopy images in the XZ plane showed arch-shaped lines resulting from a uniformly shaped melt pools during the process (Figure 4a) as well as visible laser trajectories in the XY plane (Figure 4b). This macrostructure is typical for alloys produced by LPBF [29]. The mean width of the melt pool on the etched specimens was measured as part of the macrostructural evaluation. The mean width of the melt pool was determined to be 88.5 μm for the RT specimen, 101.7 μm for 200 °C, and 123.4 μm for the specimen preheated at 400 °C. Figure 5a,b shows the details of the microstructure of the RT and specimens preheated at 400 °C in the XZ plane. The observed microstructure was characterised by a very fine cellular/dendritic structure with columnar grains in several melt pools elongated along the build direction. Some melt pools showed the presence of columnar grains growing in the direction of the thermal gradient (parallel to the build direction). Several columnar grains pass through a few melt pools (Figure 5b), which leads to strong bonding between the individual layers [11].

**Figure 6:**
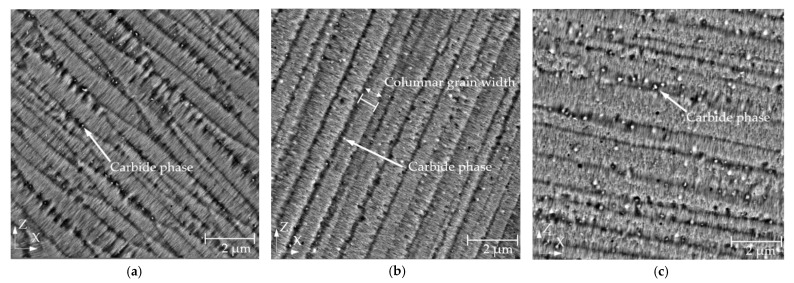
SEM-BSE microstructure of IN939 specimens fabricated at different preheating temperatures showing a gradual increase of the size and amount of the carbide phase (white dots). (**a**) XZ plane of the RT specimen; (**b**) XZ plane of the specimen with a base plate preheated at 200 °C; (**c**) XZ plane of the specimen with a base plate preheated at 400 °C.

**Figure 7:**
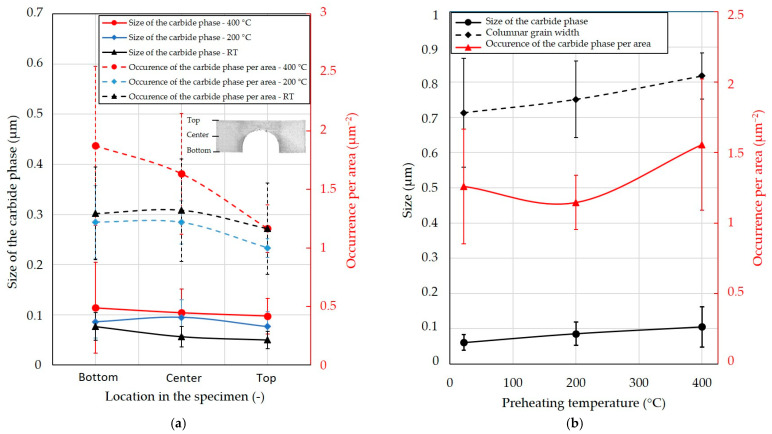
The effect of location in the specimen and preheating temperature on microstructure properties of IN939 fabricated at different preheating temperatures. (**a**) Effect of location in the specimen on the size and occurrence per area of carbide phase. (**b**) Effect of preheating temperature on the columnar grain width, size, and occurrence per area of the carbide phase.

Figure 5 contains enlarged microstructure images that were used to measure the width of columnar grains. The columnar grain width is also shown in Figure 6b. Enlarged SEM images were taken near the upper and bottom surfaces of the etched BCM specimens. No clear differences were detected in the upper and bottom locations. Therefore, the mean values were used. Measured data showed that the preheating temperature leads to wider columnar grains. The mean columnar width increased from 0.71 ± 0.15 μm when RT was used as a preheating temperature to 0.75 ± 0.11 μm for 200 °C and to 0.82 ± 0.07 μm for 400 °C (Figure 7b). 

The results of the metallographic analysis of the specimens fabricated under different preheating conditions showed significant differences in the amount and size of the carbide phase present (Figure 6). 

For all specimens, the most frequent occurrence of the carbide phase was observed near the base plate (Figure 7a). Its amount and size continuously decreased toward the surface of the specimens. The most significant decrease in the occurrence of the carbide phase per area was in the specimen fabricated using 400 °C preheating, where it decreased from 1.87 ± 0.68 μm^−2^ at the bottom to 1.17 ± 0.20 μm^−2^ at the top. That is, a decrease of about 37.8%. At preheating at 200 °C the occurrence of the carbide phase per area decreased from 1.22 ± 0.31 μm^−2^ about 18.1% to 1.00 ± 0.08 μm^−2^ at the top, while when RT was used for fabrication, the drop was 10.0% from 1.29 ± 0.40 μm^−2^ at the bottom part of the BCM specimen to 1.16 ± 0.39 μm^−2^ at the top.

The size of the carbide phase decreased in the case of preheating at 400 °C from 0.114 ± 0.091 μm at the bottom to 0.097 ± 0.035 μm at the top, which is a decrease of 14.6%. In case of preheating at 200 °C, the size of the carbide phase decreased from 0.086 ± 0.033 μm at the bottom to 0.077 ± 0.030 μm at the top, which is a decrease of 11.0%. The largest decrease in the size of the carbide phase was observed at RT. In that case, the decrease was about 35.2% from 0.077 ± 0.027 μm at the bottom to 0.050 ± 0.017 μm at the top.

The preheating led to an increase in the mean size and occurrence of the carbide phase (Figure 7b). The mean size of the carbide phase increased from 0.061 ± 0.022 μm at RT to 0.086 ± 0.033 μm at preheating at 200 °C and 0.105 ± 0.058 μm at preheating at 400 °C. 

The occurrence of the carbide phase also increased with the preheating temperature of 400 °C to 1.56 ± 0.47 μm^−2^ compare to RT (Figure 7b). However, at preheating at 200 °C, the occurrence of the carbide phase first dropped to 1.15 ± 0.19 μm^−2^ compared to RT, where 1.26 ± 0.41 μm^−2^ was measured.

### 3.3. Mechanical Properties

**Figure 8:**
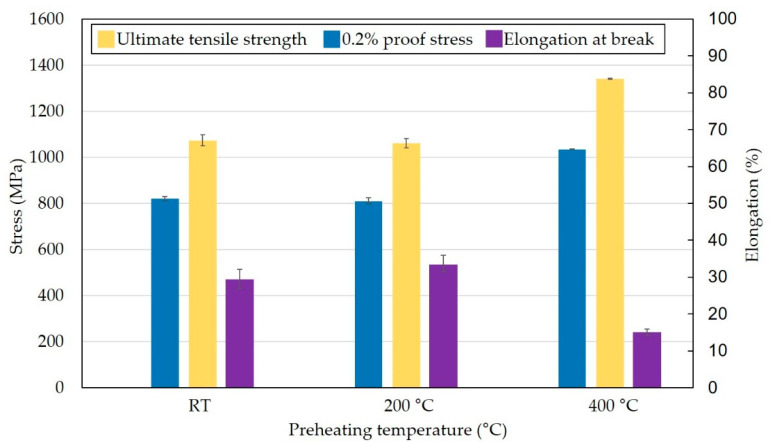
IN939 tensile properties of the specimens produced using RT and base plate preheating at 200 °C and 400 °C.

Figure 8 shows tensile properties of IN939 specimens as a function of the preheating temperature. The tensile properties did not change significantly compared to the RT and 200 °C. At RT the measured ultimate tensile strength, 0.2% proof stress, and elongation at break were 1074 ± 23 MPa, 821 ± 9 MPa, and 29.4 ± 2.6%, respectively, while at 200 °C, the properties were 1062 ± 21 MPa, 810 ± 12 MPa, and 33.4 ± 2.2%, respectively. However, when preheating was applied at 400 °C, the ultimate tensile strength and 0.2% proof stress increased significantly to 1341 ± 2 MPa and 1035 ± 1 MPa, while elongation at break dropped to 15.1 ± 0.9%. The preheating temperature of 400 °C compared to RT led to an increase in the ultimate tensile strength and 0.2% proof stress by 25% and 26%, respectively, while the elongation at break was reduced by 48.8%.

**Figure 9:**
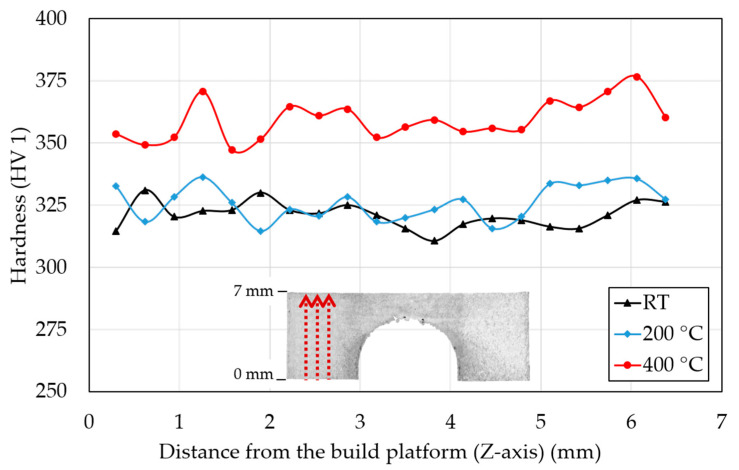
IN939 hardness of the BCM specimens produced using RT and base plate preheating at 200 °C and 400 °C.

Figure 9 shows the mean of measured hardness values in the three lines in the direction of the *Z*-axis on the BCM specimens. Specimens that were fabricated at RT and preheated to 200 °C showed very similar hardness values that in the mean value was 321 ± 26 HV 1 and 326 ± 19 HV 1. The specimen with the base plate preheated at 400 °C had a significantly higher mean hardness value of 359 ± 22.5 HV 1 that is an increase of 11.8% compared to RT. The hardness values had an approximately constant character measured on the *Z*-axis (building direction). However, the hardness near the top surface increased for all BCM specimens.

### 3.4. Effect of Powder Bed Preheating on Unfused Powder

**Figure 10:**
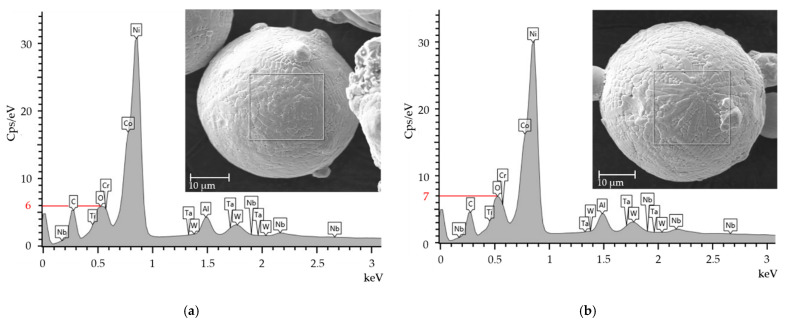
EDX analysis of the IN939 powder before and after using at high-temperature preheating. The white rectangle on powder grains symbolise the measured area. (**a**) Unused virgin powder; (**b**) used powder with the base plate preheated at 400 °C for 2 h.

**Figure 11:**
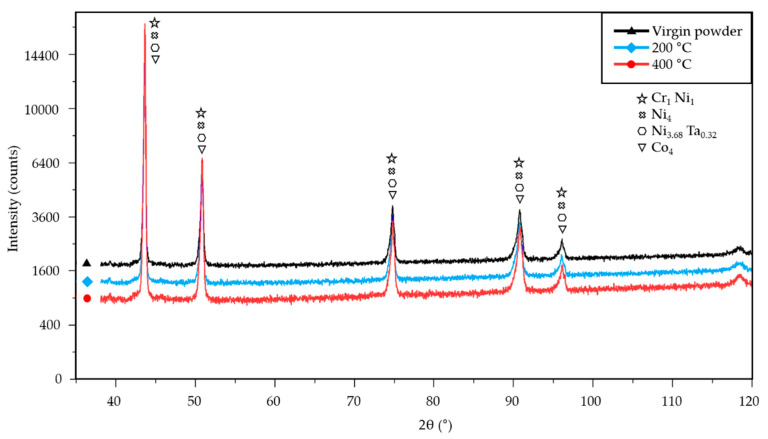
XRD analysis of the IN939 unfused powder in the three conditions: unused virgin powder, powder used at 200 °C, and powder used at 400 °C.

The EDX and XRD were used to determine the influence of high-temperature preheating on the unfused powder. Powder specimens were taken directly from the base plate after each production run. The EDX analysis of the unfused powder revealed that the effect of base plate preheating on the main elements of the chemical composition was negligible (Table 1 and Table 3). However, the intensity peaks showed an increased amount of oxygen in the unfused powder within the production run using 400 °C base plate preheating (Figure 10b) compared to the virgin powder (Figure 10a). The EDX analysis was performed with the same settings for detection figures as a comparative method between the two powder conditions and, in this case, cannot be expressed qualitatively. Furthermore, no oxides were detected in the unfused powder during the XRD analysis (Figure 11). Therefore, it can be assumed that preheating the base plate to 400 °C led to increased oxidation of the unfused powder, but the quantity of oxides did not exceed the XRD detection limit of 2 wt. %. No powder agglomerations were detected in the SEM images.

## 4. Discussion

The deformation results introduced in the previous section showed that a higher preheating temperature resulted in a higher deformation of the BCM specimens. Maximum deformations increased compared to RT and preheating of 200 °C and 400 °C to 0.01 mm and 0.04 mm, respectively, which are increases of 4.2% and 16.2% (Figure 3). The results showed that higher preheating temperatures led to higher deformations. It should be noted that the deformation results for each preheating temperature are composed of two specimens fabricated in the 0° and 90° directions according to the recoating direction of the powder. Specimens in both directions had a similar behavior (Table 2). The difference in the top surface distortion of the specimens produced in the 0° and 90° directions was 0.1 mm. This is a significant value and is attributed to the rotation of the meander scanning strategy. The limitation window parameter set in the scanning strategy caused the vectors along the gas flow direction to be skipped, resulting in an overall reduction in the length of the vectors on specimens oriented in the transverse direction (90°). Therefore, the measured distortions were lower.

The values of the top surface distortions are in contradiction with the results for materials such as titanium and aluminum alloys [15,22]. RSs of nickel-based alloy IN718 were studied under preheating conditions in the range of (50–150) °C [30]. The results showed that the higher preheating temperature led to decrease of σ_z_ residual stresses by 22%. However, the σ_x_ stresses increased from 68 MPa to 77 MPa, which is an increase of 13%. Mirkoohi et al. [23] experimentally validated that RS of IN718 gradually decreases when preheating up to 200 °C is applied [23]. However, he also predicted through simulation that preheating temperatures in the range of (250–500) °C will increase RS. The increase in RS in the study [23] was explained as a consequence of the reduced yield strength due to the coarsening of the grains. The specimens in study [23] and also in this paper were produced with the same laser-related process parameters, and powder bed preheating was the only parameter that was changed. Therefore, it can be assumed that excess heat accumulated in the manufactured specimens. 

The impact of base plate preheating on relative density was not detected and all specimens showed relative density close to 100%. The width of the melt pool increased by 35 μm when comparing the RT and the preheated specimens at temperature of 400 °C. These results correspond to the study of IN718, which showed that a larger width of the melt pool and a larger melt volume can be expected with a higher preheating temperature [23].

The microstructure studied in the etched state consists of a typical microstructure of melt pools observed in LPBF processed specimens [11,28]. The SEM analysis showed a very fine cellular/dendritic microstructure with columnar grains in all cases. The very fine cellular structure is the result of the high solidification rate due to the rapid scanning of the laser beam [29]. Columnar grains (elongated along the build direction) grew during crystallisation of the material. Several columnar grains passed through a few melt pools, leading to a stronger bond between the layers [31,32]. The frequency of occurrence of the columnar grains decreased with increasing preheating temperature. The specimens with higher preheating had a lower occurrence of columnar grains, which may be due to the reduction of the thermal gradient. The IN718 study showed that preheating at 150 °C reduces the thermal gradients, thus affecting the orientation of fine grains. The grains, checked using electron backscatter diffraction analysis, were also more randomly oriented and smaller by 19 μm under preheating temperature of 150 °C compared to 50 °C [30]. The decrease in the thermal gradient can also contribute to the columnar-to-equiaxial transition [33]. Thus, the occurrence of columnar grains in this study of IN939 decreased. However, reduced thermal gradients lead to a slower cooling rate and can increase cell spacing [34]. The mean columnar grain width of IN939 increased by 16% compared to the RT and the preheating temperature of 400 °C (Figure 7b). Variation in microstructure directly affects the mechanical properties of the material [35,36]. However, the mechanical properties of the IN939 are also influenced by evolution of the carbide phase.

The tensile test showed an increase in 0.2% proof stress and ultimate tensile strength by 26% and 25%, respectively, while elongation at break was reduced by 48.8% when comparing RT and preheating at 400 °C (Figure 8). However, the tensile properties were studied in specimens fabricated in horizontal direction. Vertically fabricated specimens can exhibit different behavior when mechanical properties depend on microstructure, which in LPBF technology is strongly influenced by building direction. 

The trend of mean hardness values corresponds to the distortion and mechanical properties of the measured specimens. RT and preheating at 200 °C did not cause significant change. However, preheating at 400 °C increased the mean hardness of the BCM specimens to 359 ± 22.5 HV 1, which is higher by 11.8% compared to RT. The hardness was measured in the building direction through the whole BCM, which showed a nearly constant character. However, the values near the top surface increased. Hardness measurement is affected by RS [37]. In additively manufactured IN718 and 316 L stainless steel, RS was detected to be higher near the top of the specimens [23,38] due to the absence of reheating from the fuse of the upper layers. This zone is called the transition area between the outer and inner surfaces [38]. Therefore, the increase in hardness values near the top surface can be explained by the increase in RS [23]. 

The mechanism that plays a significant role in the mechanical properties of nickel-based alloys is its strengthening, which is usually acquired by precipitation-hardening heat treatment. A higher preheating temperature leads to a longer solidification time of the material [39]. It can be assumed that with a longer crystallization time, the percentage of γ’ hardening precipitates and carbides, which affect mechanical properties, increases [30,40,41]. Precipitates influence the mechanical properties of the IN939 alloy [11], and high-temperature preheating can lead to its evolution during fabrication. The precipitates were detected by SEM-BSE images where they appeared as white dots formed mainly between grains (Figure 6). Based on location, contrast, and size, they should be composed of MC-type carbides [12,42], which was in additively manufactured IN939 confirmed by transmission electron microscopy [41]. Their size and occurrence gradually increased together with preheating. The average carbide size and its occurrence per area increased by 72.4% and 23.6%, respectively, when comparing RT and preheating at 400 °C (Figure 7b). Furthermore, a significant effect of location on the specimens on the carbide phase was detected. As the distance from the building plate increased, the size and occurrence of the carbide phase decreased for all preheating temperatures. The most significant effect was observed when preheating at 400 °C was used. The average grain size and occurrence per area decreased by 14.6% and 37.8% compared to the bottom and upper part of the specimen. This result shows that the build time had a significant effect on the microstructure of the material and, thus, the mechanical properties.

The resulting specimens exhibit higher hardness, ultimate tensile strength, and 0.2% proof stress but lower elongation at break. The different amounts and sizes of the carbide phase in the individual specimens fully correspond to the results of the hardness, tensile test, and deformations caused by the RS. The different temperature gradient in samples with different heights of preheating of the build plate resulted in different kinetics of the precipitation processes associated with the formation of a completely different crystallographic lattice of the carbide phase. Subsequently, precipitate formation is the cause of the increase in the internal RS level of the material.

EDX and XRD analyses showed that a higher preheating led to a slight increase in the quantity of oxides in the unfused powder. However, the oxide content did not exceed the minimum detectable value of 2 wt. % of the XRD method. The oxidation of the unfused powder does not directly affect the oxide content in the bulk material [24].

## 5. Conclusions

In this study, the effects of high-temperature preheating on deformation and indirectly on the residual stress, macrostructure, microstructure, mechanical properties, and properties of the unfused powder of Inconel 939 were investigated. The main conclusions are as follows:

- Higher preheating of the base plate led to an increase in the deformation of the top surface of the bridge curvature method specimens and thus to a higher residual stress. The increase in internal residual stress corresponds to the intensity of the precipitation process in the carbide phase.
- The higher temperature of the base plate resulted in a larger melt pool, increased columnar grain width, and increased the amount and size of the carbide phase.
- The use of higher preheating temperatures led to an increase in the hardness, ultimate tensile strength, and 0.2% proof stress but decreased the elongation at break due to an increase in the amount and size of the carbide phase.
- The build time had a significant effect on the formation of precipitates when high-temperature preheating was used.
- Rapid oxidation of unfused powder was not detected with the EDX and XRD methods.

## Figures and Tables

**Table 1:** Material supplier specification and EDX measurement of chemical composition of virgin IN939 powder in weight percentage (wt. %).

	Ni	Cr	Co	Ti	W	Al	Ta	Nb
Material supplier	balance	22.20	18.86	3.65	2.04	1.92	1.43	1.00
EDX measurement	51.57 ± 0.75	20.00 ± 0.40	18.23 ± 0.90	3.83 ± 0.45	1.83 ± 0.15	1.63 ± 0.15	1.60 ± 0.10	0.83 ± 0.15

**Table 2:** Maximum value of the deformation of the top surface as a function of the orientation of the specimen on the build platform.

	90° (mm)	0° (mm)
RT	0.19	0.29
Base plate preheating of 200 °C	0.20	0.30
Base plate preheating of 400 °C	0.23	0.33

**Table 3:** Chemical composition of IN939 unfused powder used at 200 °C and 400 °C preheating temperatures measured using EDX in weight percentage (wt. %).

	Ni	Cr	Co	Ti	W	Al	Ta	Nb
Base plate preheating of 200 °C	52.03 ± 0.30	19.60 ± 0.25	18.40 ± 0.55	3.80 ± 0.20	1.83 ± 0.20	1.53 ± 0.15	1.43 ± 0.15	0.93 ± 0.15
Base plate preheating of 400 °C	51.77 ± 1.00	19.67 ± 0.40	17.93 ± 0.60	3.73 ± 0.10	1.97 ± 0.10	1.67 ± 0.10	1.47 ± 0.15	0.83 ± 0.20

## Abbreviations

AM—additive manufacturing; BCM—bridge curvature method; EBM—electron beam melting; EDX—energy-dispersive X-ray spectroscopy; IN939—Inconel 939; IN718—Inconel 718; LPBF—laser powder bed fusion; PBF—powder bed fusion; RS—residual stress; RT—room temperature; SEM—scanning electron microscope; XRD—X-ray diffraction.
